# The complete chloroplast genome of *Abies georgei* Orr var*. smithii*, a species endemic to the Qinghai-Tibet Plateau, China

**DOI:** 10.1080/23802359.2020.1780981

**Published:** 2020-06-24

**Authors:** Jiang-Rong Li, Jia-Rui Chen, Jian-Ke Wang, Wei-Lie Zheng

**Affiliations:** a Institute of Tibet Plateau Ecology, Tibet Agriculture and Husbandry University, Nyingchi, China; b Key Laboratory of Forest Ecology in Tibet Plateau (Tibet Agriculture and Animal Husbandry University), Ministry of Education, Nyingchi, China

**Keywords:** Coniferous, Pinaceae, plastid genome, phylogeny

## Abstract

*Abies georgei* Orr var. *smithii* is an evergreen coniferous species of Pinaceae, and is endemic to the Qinghai-Tibet Plateau of China. Considering its vital ecological functions in this unique area, the complete chloroplast (cp) genome was constructed in this study to provide genetic information for its further study of conservation and evolution. The complete cp genome is 121,213 bp in length with GC content of 38.3%, and contains a tetrad structure, including a large single copy region of 76,278 bp, a small single copy of 42,575 bp, and two very short repeats of 1,180 bp for each. Besides, it contains 113 genes in total, including 74 CDSs, 35 tRNAs, and four rRNAs. This genome has been deposited in Genbank under accession number of MT527722.

*Abies georgei* Orr var. *smithii* is an evergreen coniferous species of Pinaceae, and is endemic to the Qinghai-Tibet Plateau (QTP) of China at an altitude of 2500–4400 meters. *A. georgei* var. *smithii* trees are dominant at alpine timberline in southeast of the QTP. As these trees grow under the harsh and unique habitats, they have important ecological functions and are sensitive to climate change, soil and water conservation and biodiversity maintenance (Guo and Zhang 2015; Guo et al. 2016). However, habitat destruction and excessive logging have led to a dramatic decline in the number of the tree resources. Thus, conservation of the genetic resources is urgently needed at present. However, because of its unique and remote distribution area, lacking of genomic information greatly restricts the genetic conservation of the valuable resources.

Plant chloroplast (cp) contain cp genome is independent of the nuclear genome, and inherited maternally. Due to its length is general short, together with the structure and sequences are conserved, the cp genome could provide ideal genetic information for further population genetic studies (Li et al. 2019). Therefore, the completed chloroplast genome of *A. georgei* var. *smithii* was constructed in this study (Figure 1).

**Figure 1.**
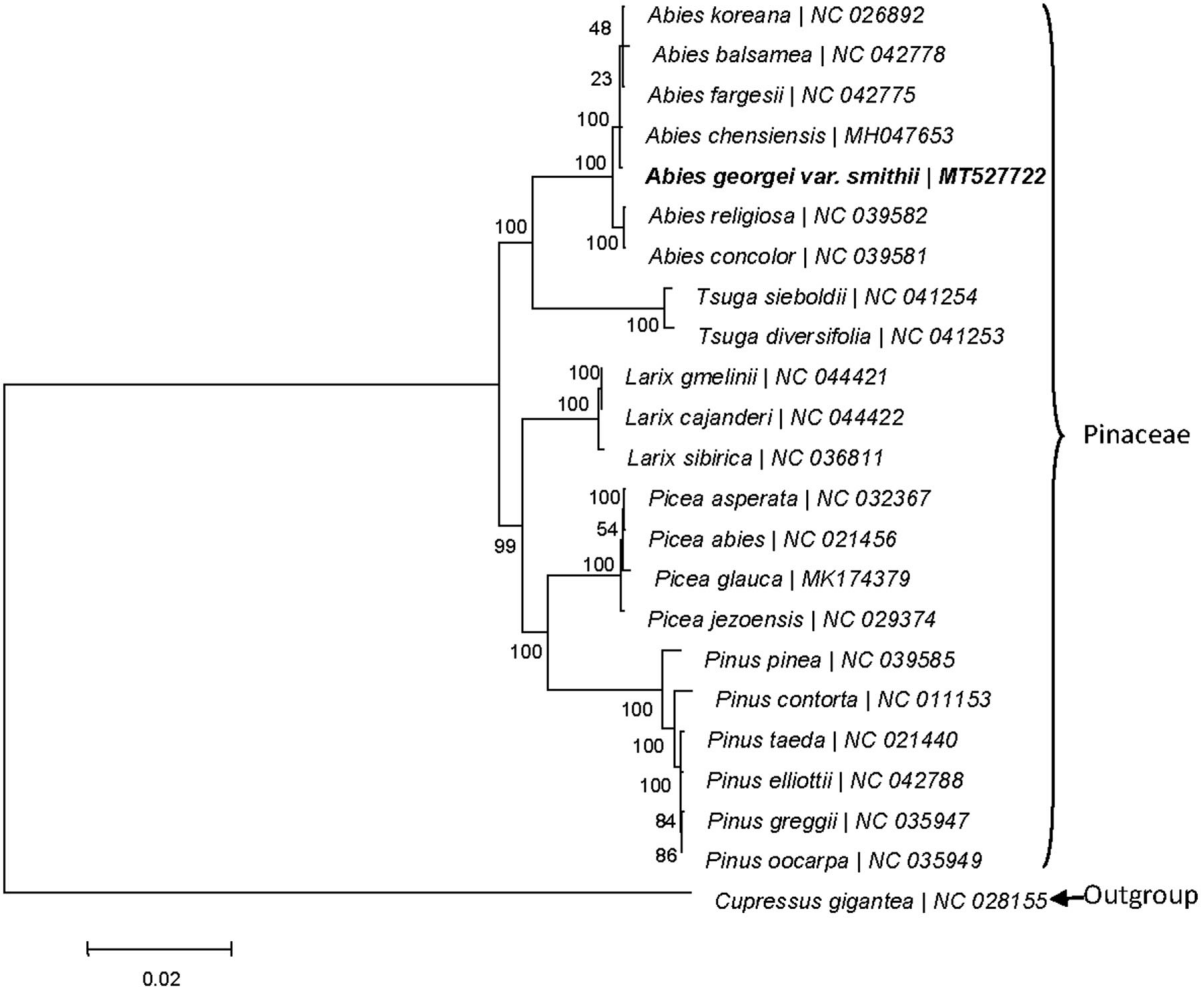
Phylogenetic tree based on the maximum-likelihood (ML) of 23 completed cp sequences. The bootstrap value based on 1000 replicates is shown on each node.

The needle-leaves of *A. georgei var. smithii* were collected in Sygera Mountain located in southeast Tibet, China (29°36′58.03″ N, 94°41′41.15″ E, 4380 m H). The corresponding voucher specimen (Ag012Jiangrong) was deposited in the herbarium of Institute of Plateau Ecology, Tibet Agriculture & Animal Husbandry University, China. Total genomic DNA was isolated from the leaves by using modified CTAB method (Healey et al. 2014). DNA library construction and high-throughput sequencing on Illumina HiSeq X Ten platform were performed in Novogene Inc. (Beijing, China). After sequencing, raw data were trimmed and filtered to obtain clean reads by using Trimmomatic v0.36 (Bolger et al. 2014). De novo assembly of contigs was done by using CLC Genomics Workbench v8 (CLC Bio, Denmark), resulted contigs were filtered with reads coverage over 20. Among the filtered contigs, those length above 10,000 bp were blast against nucleotides database of NCBI, and three of them which have high identity with cp genome of *A. chensiencis* (MH047653) (Zhao et al. 2019), were picked. Among them, the longest one with length of 76,307bp had the highest homology of 99.6% with cp genome of *A. chensiencis*. Thus, the cp genome of *A. chensiencis* was used as a reference for subsequent assembling of that of *A. georgei var. smithii* using MITObim v1.7 (Hahn et al. 2013) with the clean reads. The assembled cp genome was verified and corrected with the above three cp contigs assembled de novo. Gene annotation was performed using Geneious (Biomatters Ltd., Auckland, New Zealand) (Kearse et al. 2012) by comparing with the reference genome, and manual checking and adjustment were adopted. A Neighbor-joining tree was constructed based on 55 homologous protein coding sequences (CDSs) of 22 cp genomes from Pinaceae species and one from Cupressaceae species with a bootstrap value of 1000.

The constructed cp genome of *A. georgei var. smithii* is deposited in Genbank under accession number of MT527722. It is 121,213 bp in length with GC content of 38.3%, and contains a tetrad structure, including a large single copy region of 76,278 bp, a small single copy of 42,575 bp, and two very short repeat regions of 1,180 bp for each. Besides, it contains 113 genes in total, including 74 CDSs, 35 tRNAs, and four rRNAs.

Phylogenetic analysis based on the homologous CDSs from 23 cp genomes showed that all species of Pinaceae clustered together, among them, *A. chensiencis* is the closest to *A. georgei* var. *smithii* with a bootstrap value of 100%. Therefore, the construed cp genome of *A. georgei* var. *smithii* would provide valuable genetic information for the further study for its conservation and evolution.

## Data Availability

The data that support the findings of this study are openly available in GenBank of NCBI at https://www.ncbi.nlm.nih.gov, reference number MT527722.
